# Explanatory argumentation in natural language for correct and incorrect medical diagnoses

**DOI:** 10.1186/s13326-024-00306-1

**Published:** 2024-05-30

**Authors:** Benjamin Molinet, Santiago Marro, Elena Cabrio, Serena Villata

**Affiliations:** Université Côte d’Azur, CNRS, Inria, I3S, Rte des Lucioles, Sophia Antipolis, 06900 Alpes-Maritimes France

**Keywords:** AI in medicine, Natural language processing, Information extraction, Argument-based natural language explanations, Healthcare

## Abstract

**Background:**

A huge amount of research is carried out nowadays in Artificial Intelligence to propose automated ways to analyse medical data with the aim to support doctors in delivering medical diagnoses. However, a main issue of these approaches is the lack of transparency and interpretability of the achieved results, making it hard to employ such methods for educational purposes. It is therefore necessary to develop new frameworks to enhance explainability in these solutions.

**Results:**

In this paper, we present a novel full pipeline to generate automatically natural language explanations for medical diagnoses. The proposed solution starts from a clinical case description associated with a list of correct and incorrect diagnoses and, through the extraction of the relevant symptoms and findings, enriches the information contained in the description with verified medical knowledge from an ontology. Finally, the system returns a pattern-based explanation in natural language which elucidates why the correct (incorrect) diagnosis is the correct (incorrect) one. The main contribution of the paper is twofold: first, we propose two novel linguistic resources for the medical domain (i.e, a dataset of 314 clinical cases annotated with the medical entities from UMLS, and a database of biological boundaries for common findings), and second, a full Information Extraction pipeline to extract symptoms and findings from the clinical cases and match them with the terms in a medical ontology and to the biological boundaries. An extensive evaluation of the proposed approach shows the our method outperforms comparable approaches.

**Conclusions:**

Our goal is to offer AI-assisted educational support framework to form clinical residents to formulate sound and exhaustive explanations for their diagnoses to patients.

## Introduction

Explanatory Artificial Intelligence (XAI) is a main topic in AI research nowadays, given, on the one side, the predominance of black box methods, and on the other side, the application of these methods to sensitive scenarios like medicine and law. One of the main reasons why making AI solutions *explainable* and *trustable* lies in the law. With the AI Act, for instance, the European Commission regulates the use of “high-risk artificial intelligence” [1] for the medical domain, discussing sensitive topics like explainability, responsibility and gouvernance. Among the huge set of contributions in this area [2, 3], some approaches highlight the need to build explanations which are sound and clearly interpretable, leading to the investigation of the generation of argument-based explanations [4]. These explanations are intended to be not only rational, but “manifestly” rational [5], such that arguers can see for themselves the rationale behind the inferential steps taken. This is particularly important when explanations are used for educational purposes, like to form students to present the elements which conducted them to take a certain decision in a clear way. Again, in sensible scenarios like medicine and law this is a mandatory requirement, e.g., clinical residents need to explain grounding on evidence how they come up with a certain diagnosis, and law students need to explain how they build a certain deliberation based on the law and the previous cases. Although this task is already challenging when dealing with formal representation of the explanations [6, 7], it becomes even more challenging if we target the generation of natural language argument-based explanations [4, 8]. We refer the reader to Tran et al. (2021) for a systematic overview of existing research on healthcare recommender systems, highlighting the complexity and variety of approaches in this domain.

In this paper, we address this open research challenge in the medical domain. More precisely, we answer the following research question: how can we generate fine-grained natural language explanations for correct and incorrect diagnoses?

To answer this research question, we rely on a corpus of training data for med residents. These students are trained through tests which consist of a textual description of a clinical case (i.e., symptoms experienced by the patient, results of clinical exams and analysis, findings and vital signs, and some further information concerning the patient herself like the age), and then the following question “Which of the following is the most likely diagnosis?” arises. The test is composed of a number of possible answers to this question, i.e., potential diagnoses, among which one of them is the correct diagnosis and the others are incorrect. The solution consists in selecting the correct answer. To ensure the effectiveness of this approach, cognitive and epistemological criteria are proposed in Explainable Artificial Intelligence (XAI), as discussed by Cabitza et al. (2023). In order to generate automatically natural language explanations about why the correct and incorrect diagnoses are so, we rely on three main modules: first, we identify the symptoms and the findings in the textual description of the clinical case, secondly we align the detected symptoms with the concepts in the verified medical ontology called Human Phenotype Ontology (HPO) in order to find what are the diseases associated to these symptoms, and finally, we convert the detected findings into medical terms considering their biological boundaries (e.g., “temperature over 38 degrees” can be converted into “fever”). Given all the retrieved knowledge pieces, we automatically generate explanations of the kind: “The patient is affected by [$diagnosis_x$] because the following relevant symptoms have been identified: [correct diagnosis symptoms and converted findings]. The [$diagnosis_y$] is incorrect because the patient is not showing the symptoms [incorrect diagnosis symptoms]. Furthermore, symptoms [correct diagnosis missing frequent symptoms] are also frequent symptoms observed for [$diagnosis_x$] and could not be found in the clinical case”.

The main contribution of the paper is twofold: first, we present two novel linguistic resources for the medical domain, i.e., the MEDQA-UMLS-Symp dataset which contains a set of clinical case descriptions together with a set of possible questions and answers on the correct diagnosis from MedQA [9], annotated with medical entities from UMLS [10], and a database of biological boundaries for common findings; second, we introduce a complete pipeline to generate natural language explanations for correct and incorrect diagnosis, relying on clinical entities detected from clinical cases and aligned with medical ontologies. This journal paper extends our previous contribution [11] showing that medical findings and vital signs can enhance explanations by converting observed values to a medical terminology, based on a manually verified medical database and large Language Models, and enriching explanations with findings information.

The work presented in this paper is, to the best of our knowledge, one of the very few examples of full framework to automatically generate natural language explanations in medicine. Given the sensibility of the medical scenario, we rely on an hybrid AI approach mixing both symbolic AI (i.e., the ontology alignment with the HPO ontology, and pattern-based approach to generate the explanations) and numerical AI (i.e., the Large Language Models and generative AI). This approach allows us to obtain good results (outperforming standard baselines and competing approaches) always ensuring the verification of the medical knowledge employed in the generated evidence composing the explanations.

The paper is organised as follows. In Background section, we present the existing approaches in the literature for information extraction on medical text and medical term ontology alignment, and we compare them with the proposed approach. In Resources section, we introduce the two linguistic resources we built and how we assess the reliability of these resources. Methods section presents the proposed pipeline for natural language explanation generation and details the different modules composing it. In Experiments section, we present the experimental setting, we report on the obtained results and we address an error analysis. In Generating Natural Language Explanations section, we introduce the patterns for generating the explanations both for correct and for incorrect diagnoses. A discussion and conclusion end the paper.

## Background

Artificial intelligence (AI) has become a lever for progress in the medical field. This field deals with different sources of information such as images, analysis records, biomedical data and text in natural language. Focusing on the latter, ongoing research projects are investigating how to automate diagnosis prediction using unstructured text [12]. Previous approaches have focused on building rule-based systems to predict diagnoses [13, 14] wheras, more recently, approaches based on machine learning methods have been developed to predict breast cancer [15], psychiatric conditions [16], and HIV risks [17]. As another example, Bracchi et al. [18] propose a system based on RNN and CNN to predict the cerebrovascular cause of transient ischemic attacks. While many studies attempt to predict medical diagnoses, very few seek to explain them [19]. This section presents and discusses existing work on the generation of natural language explanations and the role of verified sources of medical knowledge such as ontologies and thesauri in generating such explanations. We will first present available datasets, then we will describe existing approaches aligning medical entities with ontologies, and finally we will focus on the existing methods to generate natural language explanations.

### Medical data and linguistic resources

A considerable amount of research effort focused on the construction of robust and trustworthy sources of knowledge like ontologies and vocabularies. Several of these resources are centered around clinical terms, such as SNOMED CT [20]^1^ and ICD-10 codes [21], while others focus on more specific areas. RxNorm [22], for instance, is devoted to clinical drugs, CPT [23] is built around procedural terminology, and MeSH [23] is designed for cataloging and searching biomedical information. The Human Phenotype Ontology (HPO) [24] provides a comprehensive platform for discussing human phenotypic abnormalities. Furthermore, Bodenreider [10] proposed a Metathesaurus based on the aforementioned vocabularies and many others, into a unified structure. This integrated resource includes names, relationships, attributes, and other details related to biomedical and health-related concepts.ad

In parallel to the efforts made in creating reliable medical vocabularies, significant advancements have been made in the compilation of medical datasets, particularly in natural language. Notably, this has been accomplished through shared tasks such as i2c2 (renamed as n2c2) for Information Extraction (IE) [25, 26], MEDIQA (QA) used in Natural Language Inference (NLI), Recognizing Question Entailment (RQE), and Question Answering [27], and SemEval 22 with IE and NLI tasks [28–30].

While Johnson et al. [31] proposed the MIMIC-III dataset consisting of textual data about vital signs, medications, laboratory measurements, observations and more, other efforts have focused both on structured and unstructured data. For instance, eICU and PhysioNet[32, 33] are two contributions that have been key in enhancing the body of available medical datasets by collecting respectively anonymized structured data from patients (including vital sign measurements, care plan documentation, diagnosis information, treatment information) and signals archive. Simultaneously, resources like the UK Biobank and the Cancer Imaging Archive [34, 35] include both medical images and textual data.

Numerous contributions focused on identifying medical Named Entities from article abstracts, primarily from PubMed. These approaches to Named Entity Recognition (NER) target various biomedical aspects, ranging from Part-of-Speech (PoS) tagging with the Extended GENIA dataset [36], to more detailed entity annotations on full articles, as in the CRAFT corpus [37]. The AnatEM corpus [38] and some of the BioNLP Shared Tasks [39, 40] concentrate on entities and relations, while other approaches [41–46] specifically address gene, protein, and species entities.

However, only a limited number of studies have focused on disease and medical findings annotation, e.g., the NCBI disease corpus [47] and the MEDQA-USMLE-Symp dataset [11], which is annotated with UMLS symptoms and findings tags. Despite these two resources, the issue of matching medical findings to symptoms is still an open research question. This highlights the need for further research in this area to improve the understanding and adoption of medical findings for more accurate and comprehensive diagnostic and explanatory tasks.

### Information Extraction on medical text

Many robust off-the-shelf pipeline toolkits like Spacy [48], MedSpacy [49], and CLAMP [50], have been recently proposed for text processing, and in particular, to process medical text. Notably, MedSpacy is a specialized extension of Spacy, custom-built for clinical language processing. CLAMP stands out due to its capability for NER and its interactive interface for annotating clinical text. However, their rule-based approach for NER in the medical domain makes it complex to apply it to named entities not originally considered in the tool, and new rules need to be defined.

Recent approaches cast NER as a sequence labeling task, where transformer-based models have shown remarkable performance, especially when fine-tuned on specific domains. Naseem et al. [51] showed that pre-training the ALBERT model on a large-scale biomedical corpus enhances the model’s ability to capture the context found in biomedical NER tasks. This specialized approach has resulted in these models outperforming non-specialized counterparts and achieving top-tier results on several datasets. BioELECTRA [52] exemplifies this trend by pre-training a biomedical language model using biomedical text and vocabulary with the ELECTRA architecture. Other BERT-based models, such as SciBERT [53], BioBERT [54], PubMedBERT [55], and BioMed-RoBERTa [56], which is based on RoBERTa, have also been designed for the biomedical domain.

Other approaches like UmlsBERT [57] integrate domain knowledge from the Unified Medical Language System (UMLS) ontology into a contextual embedding model. The model’s strength lies in its ability to associate different clinical terms with similar meanings in the UMLS knowledge base, creating meaningful input embeddings by leveraging information from the semantic type of each word. Our study compares the representation of symptoms found in clinical cases with different contextual embeddings, seeking to identify a representation that aligns with the one provided in the Human Phenotype Ontology [24].

Finally, Raza et al. [58] propose the Bio-Epidemiology-Ner (BioEN) pipeline, an approach inspired by [11], where they fine-tune a DistilBERT [59] model, a simplified and more computationally efficient version of BERT, for the task of biomedical NER. They adapt the last layer of the pre-trained DistilBERT model to their specific biomedical task and adjust the input and output dimensions accordingly. However, the labels they use are not derived from any certified ontology or medical source, making this approach ad hoc to their NER labels and limiting its reusability. Furthermore, their approach does not account for the broader scope of medical findings, which include vital signs and analysis results, essential elements to analyse clinical cases.

### Medical term alignment

In our work, we not only aim to detect symptoms from clinical cases but also to align them with medical ontologies such as the Human Phenotype Ontology (HPO). Such alignment is required to elucidate the connections between symptoms, that can be expressed in layperson terms, and diseases. Manzini et al. [60], for instance, propose an automated system for translating layperson terminology to HPO. This system leverages a neural network and a vector space to generate and compare vector representations of medical and layperson terms. The main limitation of this approach is that it translates layperson terms without considering the context, potentially missing relevant information that may change the semantics of the term. In contrast, the approach we propose in this paper accounts for the context in which the layperson term is used, thus enabling a more accurate mapping to an HPO term.

In addition to symptoms, medical explanations often rely on the results of additional tests, such as blood tests and vital sign measurements. Consequently, it is crucial to take into account and interpret these data. Several recent studies focus on the automatic detection of vital signs in digitized patient records, such as Electronic Medical Records (EMR)[61] or Electronic Health Records (EHR) [62–64]. However, none of these studies, to the best of our knowledge, concentrate on training exams. These exams can be clinical cases that utilize a different structure from EMR or EHR and often show a more narrative text, presenting symptoms, patient history, and lab results as part of a broader storyline.

Earlier contributions focusing on the extraction of medical findings and vital signs proposed rule-based approaches [61, 63, 64]. Although they obtained good results, they still require specialists to create and refine the rules, limiting their generalisability to other medical tasks. In contrast, the approach proposed by Gavrilov and al. [62] employs a deep learning strategy, training a model on Russian data using Bloom’s embedding methods implemented in SpaCy.

While these works showed good performance in detecting vital signs, their applicability range remains limited. First, they primarily focus on detecting the six fundamental vital signs, i.e., blood pressure, heart rate, respiratory rate, body temperature, height, and weight. Even if these vital signs are the most used in the literature [61–64], some complementary analysis such as laboratory analysis are needed to confirm or discard a diagnosis. Since these findings are numerous and evolve with time, a rule-based system would require a large number of experts to create and maintain the rules. [64] offers a NER assigning also a quality score to each entity, computed according a set of rules for each vital signs.

### Medical explanations generation

Natural language explanation generation has received a lot of attention in recent years, grounding on the progress of generative models to train specific explanatory systems. [65] generates explanations by justifying a relation (i.e., *entailment*, *contradiction* or *neutral*) for a premise-hypothesis pair by training a Bi-LSTM on their e-SNLI dataset, i.e., the Stanford Natural Language Inference [66] dataset augmented with an explanation layer which explains the SLNI relations. [67] propose to generate short explanations with GPT-2 [68], learned together with the input by a classifier to improve the final label prediction, using e-SNLI [65]. These solutions are not applicable to the medical domain given that explaining a medical diagnosis is a sensible task which can hardly be restrained to the above-mentioned three basic relations (considered in [65] and [67]). Narang et al. [69] propose an approach based on the T5 model [70] to generate an explanation after a prediction. The problem with these approaches based on neural models is that we do not master the internal knowledge of these models, which can generate errors on the veracity of the data. Again, this solution is not applicable to the medical scenario, since explanations are required to be structured following precise argumentative structures [71–73] and to be grounded on verified medical knowledge, like the one we inject through the HPO.

Other approaches use explanations via templates [74, 75]. For instance, Abujabal et al. [76] use templates and inject the reasoning steps and query of their Q &A system. To the best of our knowledge, no related work generates natural language post-hoc explanations under the form of arguments for the medical domain.

## Resources

The availability of clinical data (both clinical cases and knowledge bases) is of crucial importance for our study. In the following, we describe one of the main contributions of this paper, i.e., the annotation of the MEDQA-USMLE Clinical Cases with labels extracted from the UMLS medical meta-thesaurus and the ontology used to explain correct and incorrect diagnoses. Furthermore, this section details the creation and assessment of the proposed medical findings with respect to the medical term database, used to interpret findings and vital signs that are subsequently integrated in our explanations. These resources, while distinct, both play a roles in our pipeline. The dataset is utilized for training our Named Entity Recognition (NER) models and the database is employed to align findings with relevant medical concepts in the Human Phenotype Ontology (HPO).

### The MEDQA-USMLE-Symp dataset

To train and evaluate the proposed approach to build natural language explanatory arguments, we rely on the MEDQA dataset [9], which contains a set of clinical case descriptions together with a set of possible questions and answers on the correct diagnosis. The questions and their associated answers were collected from the National Medical Board Examination in the USA (USMLE), Mainland China (MCMLE), and Taiwan (TWMLE). In this work, we only focus on the clinical cases and the questions in English (i.e., *USMLE*). In total, the MEDQA-USMLE dataset consists of 12,723 unique questions on different topics, ranging from questions like “Which of the following symptoms belongs to schizophrenia?” to questions about the most probable diagnosis, treatment or outcomes for a certain clinical case [9]. To reach our goal, we extract the clinical cases belonging to the latter group, which are intended to test medical residents to make the correct diagnosis. We end up with 314 unique clinical cases associated with the list of possible diagnoses.

#### Annotation of the MEDQA-USMLE Clinical Cases

To annotate the clinical cases from the MEDQA-USMLE dataset, we rely on the labels from the Unified Medical Language System (UMLS) [10] Semantic Types, making it consistent with standard textual annotations in the medical domain [77–79]. In particular, we annotate the following elements in the clinical case descriptions: *Sign or Symptom*, *Finding*, *No Symptom Occurrence*, *Population Group*, *Age Group*, *Location* and *Temporal Concept*. Among the extensive variety of labels offered by the UMLS Semantic Types, we chose these specific ones to suit our data and question type: “Diagnosing a patient’s clinical case”. Our selection of these seven labels was informed by consultations with medical experts and determined by their explanatory power in our specific context.

In this paper, we mainly focus on the *Sign or Symptom* and *Findings* labels as they offer critical insights for the diagnosing task. However, in anticipation to the development of a more fine-grained approach as future research, we have conducted a comprehensive annotation across all seven labels, allowing for future reuse and exploration of these additional dimensions. Concerning the guidelines^2^, quantifiers defining a symptom have not been annotated (e.g., when we find “moderate pain”, we only annotate “pain”). The labels *Sign or Symptom* and *No Symptom Occurrence* are associated only to the text snippet defining the symptom in a sentence. *Findings* consists of such information discovered by direct observation or measurement of an organism’s attribute or condition. For instance, *components* in “Her *temperature is 39.3*^∘^*C (102.8*^∘^*F)*, *pulse is 104/min*, *respirations are 24/min*, and *blood pressure is 135/88 mm Hg*”. *Location* refers to the location of a symptom in the human body, and *Temporal Concept* is used to tag time-related information, including duration and time intervals. *Population Group* and *Age Group* highlight information on the age and gender of the patient.

##### Example 1

Her **temperature is 37.2**^∘^**C (98.9**^∘^**F)**, **pulse is 90/min**, and **blood pressure is 122/70 mm Hg**, **test of the stool for occult blood is positive** Her **temperature is 39.3**^∘^**C (102.8**^∘^**F)**, **pulse is 104/min**, **respirations are 24/min**, and **blood pressure is 135/88 mm Hg**.

In Example 1, the entities labeled as ‘findings’ are showcased. This annotation includes the term indicating the finding, the corresponding value, and the relevant unit. Notably, we also consider test results as a part of ‘findings’. It is important to note that our annotation approach deliberately excludes quantifiers of symptoms and findings, such as ‘few’, as well as descriptive adjectives, such as ‘scattered’ or ‘large’.

##### Example 2

A previously healthy 34-year-old woman is brought to the physician because of fever and headache **for 1 week**.

##### Example 3

Menses occur at regular **28-day intervals** and **last 5 to 6 days**.

Examples 2 and 3 illustrate the annotation of temporal concepts, such as duration and time intervals. These examples highlight the necessity of annotating words like ‘for’, which indicates a time period. They also highlight the importance of capturing non-specific temporal tags, such as ‘last 5 to 6 days’, as they provide crucial context for understanding the progression and duration of symptoms or medical conditions.

To address the annotation process of the MEDQA-USMLE dataset, we first carried out a semi-automatic annotation relying on the UMLS database. We processed each clinical case through the UMLS database and obtained all the entities detected along their Concept Unique Identifiers (CUI) and their semantic type. The semantic type is then used to disambiguate the entities and generate the pre-annotated files. After the definition of the detailed annotation guidelines^3^ in collaboration with clinical doctors, three annotators with a background in computational linguistics carried out the annotation of the 314 clinical cases.

Given the complexity of determining which disease corresponds to a given case, a manual annotation was required. Identifying the appropriate disease requires deep medical knowledge, a perfect understanding of context, and familiarity with symptom synonyms since symptoms within a case can be recorded using either their medical terminology or their common jargon. Owing to the challenge of gathering a complete database of all pertinent information, we could not rely exclusively on automatic annotation.

To assist the annotators, we initiated the process with a pre-automatic annotation using the Brat visualization tool [80] together with QuickUMLS^4^, a tool that leverages UMLS data and pre-annotate them into the Brat visualization tool. This automatic annotation was then manually corrected and completed using the ontology of diseases and relevant symptoms HPO.

The annotation process involved various categories, each contributing to a comprehensive description of the data: Findings, Relevant Symptoms, Sign or Symptom, Age Group, Population Group, Temporal Concept, Location, No Symptom Occurrence, and No Finding Occurrence. This annotation schema helps to capture the complexity of the clinical cases, enabling an accurate mapping to disease categories.

To ensure the reliability of the annotation task, the inter-annotator agreement (IAA) has been calculated on an unseen shared subset of 10 clinical cases annotated by four annotators, obtaining a Fleiss’ kappa [81] of 0.70 for all of the annotated labels, 0.61 for *Sign or Symptom*, 0.94 for *Location*, 0.71 for *Population Group*, 0.66 for *Finding*, 0.96 for *Age Group* and 0.96 for *No Symptoms Occurrence*. We can see a substantial agreement for *Sign or Symptom*, *Finding* and *Population Group*, and an almost perfect agreement for *Location*, *Age Group* and *No Symptoms Occurrence*.

**Table 1:** Statistics of the MEDQA-USMLE-Symp dataset

Label	# Entities
Sign or Symptom	1579
Finding	1169
Temporal Concept	567
Location	498
Population Group	364
Age Group	304
No Symptom Occurrence	264

Table 1 reports on the statistics of the final dataset, named MEDQA-USMLE-Symp^5^. The accuracy of the annotations provided by the three annotators has been validated by a clinical doctor. Of the seven entity labels, only three contain medical vocabulary (Sign or Symptom, Finding, and No Symptom Occurrence) and they have been evaluated by this expert. More specifically, we randomly sampled 10% of the data (i.e., 30 cases) and we asked the clinician to verify whether the entity was correctly labeled and whether there were any missing or extra words. The results of the validation showed that 98% of the data was labeled correctly. Errors were distributed randomly, being the majority of them annotation errors with missing/extra letters from the labels (e.g., *itchy rash* annotated as *tchy rash* or *generalized joint pain* annotated as *generalized joint pain*). Less than 2% of the instances were evaluated as incorrectly labeled (e.g., a Finding that was labeled as a Sign or Symptom or vice versa). We also manually annotated, in our test set, the terms equivalent to the symptoms in the HPO ontology. Out of the 162 symptoms detected, 88 were aligned with the concepts in the ontology. These annotations are available within the project repository^6^.

#### Knowledge Base of Diseases and Relevant Symptoms

To collect the medical knowledge needed to define whether a detected symptom is relevant with respect to a given disease, we employ the HPO knowledge base to retrieve *(i)* the relevant information of each diagnosis which is proposed as an option to answer the question “Which of the following is the most likely diagnosis?”, and *(ii)* the symptoms (named *terms* in HPO) associated to each diagnosis. The Human Phenotype Ontology (HPO) is a comprehensive, structured vocabulary for describing phenotypic abnormalities encountered in human diseases, which is often used in genetic and rare disease research. The use of HPO rather than other resources such as SNOMED CT is justified in particular by the fact that this knowledge base includes, in addition to other knowledge, information on the frequency^7^ of the occurrence of symptoms, defined in collaboration with ORPHA^8^ as follows: Excluded (0%); Very rare (1-4%); Occasional (5-29%); Frequent (30-79%); Very frequent (99-80%). Obligate (100%); This kind of information is particularly valuable in our application scenario to generate fine-grained explanations. HPO integrates different sources of symptoms, including ORPHA and OMIM^9^. This knowledge base is quite rich and contains also (hierarchical) links between symptoms (i.e., Symptom S2 subclass of Symptom S1), genes or definitions.

### Medical findings database

While annotating entities, we observed that “Findings” represent a particularly complex category. This is because their interpretation involves two steps: i) identifying the normal boundaries, and ii) linking them to the appropriate medical term. This complexity drives us to build a specific database that encompasses the most frequently occurring medical findings within our clinical environment. These data will be used to automatically convert medical findings to medical terms.

The medical findings database is designed to facilitate the interpretation of medical test results, converting raw findings, such as “Platelet count is 100,000 platelets per microliter of blood”, into associated symptoms and medical terms, in this case, “Thrombocytopenia.” To this goal, it is necessary to determine whether a given value is classified as “high” or “low” in comparison to normal values, and subsequently associate it to a relevant medical term. In this study, we define “normal values” as those provided by known medical sources^10^ and from the MedQA-USMLE tests themselves [9], bearing in mind that these values are simplifications and do not account for different ethnicity, gender-specific findings, or age-related variations. In order to ensure the comprehensiveness of the database, to foster future reuse of the resource and to maintain compatibility with existing systems, we have also associated each medical term with corresponding medical codes from the International Classification of Diseases (ICD-10), the Human Phenotype Ontology (HPO), and the international health terminology standard SNOMED CT (July 2023 release). The International Classification of Diseases (ICD-10) is a globally recognized system for categorizing diseases and other health conditions, maintained by the World Health Organization (WHO). The international health terminology standard SNOMED CT is a comprehensive, multilingual clinical terminology system that covers a wide range of medical concepts, including diseases, symptoms, and procedures. A representative example of the final database can be found in Table 2 for low values, and Table 3 for high values.

**Table 2:** Low vital sign values and their corresponding medical terms, HPO codes, ICD-10 codes, and SNOMED CT codes for glucose and platelet count

Finding	Value	Medical term	HPO	ICD-10	SNOMED CT
Glucose (Glu)	70 mg/dL	Hypoglycemia	HP:0001943	E16. 2	271327008
Platelet count	150000 mcL	Thrombocytopenia	HP:0001873	D69. 6	74576004

**Table 3:** High vital sign values and their corresponding medical terms, HPO codes, ICD-10 codes, and SNOMED CT codes for glucose and platelet count

Finding	Value	Medical term	HPO	ICD-10	SNOMED CT
Glucose (Glu)	99 mg/dL	Hyperglycemia	HP:0003074	R73. 9	-
Platelet count	450000 mcL	Thrombocytosis	HP:0001894	D75. 83	6631009

#### Semi-automatic database creation

Developing a new knowledge resource specifically tailored for our requirements in the medical domain presents a considerable challenge, particularly given the significant manual effort and human involvement necessary to conceive, collect, align, and verify the data. Moreover, obtaining expert assistance in the medical field can be difficult due to the demanding nature of the work and the workload of medical professionals. Consequently, we propose a semi-automatic method for generating a database by harnessing the capabilities of state-of-the-art generative large language models, such as ChatGPT [82, 83], which are pretrained on huge amounts of text, including medical documents. We constrain the language model to generate knowledge in a tabular format expressed as free text, such as Markdown. This free text is subsequently parsed using regular expressions, allowing for the extraction of structured data to be incorporated into the database. This database is then refined and verified by domain experts as explained below. It is important to notice that applying a semi-automatic approach to fill in the first and basic version of the database already significantly reduces the manual effort.

To assess this semi-automatic approach we addressed both: i) an automated evaluation through comparison with an existing database, and ii) a human evaluation involving a medical expert for correction and validation of the database.

#### Automated evaluation

We first evaluate our semi-automatic approach on a similar database of laboratory reference ranges for healthy adults^11^, in order to see if such Large Language Model (LLM) would be useful for assisting the database creation. This selected database provides reference ranges for various categories such as Electrolytes, Hematology, Lipids, Acid base, Gastrointestinal function, Cardiac enzymes, Hormones, Vitamins, Tumor markers, and Miscellaneous, but does not specify the medical terms associated with these values. To evaluate our method, we used a generative language model several times on the Electrolytes category as the gold standard. Since all the values are numerical we compute the accuracy on the mean value of the multiples runs for each findings and compare with the gold. This gave us an understanding of the efficacy of LLMs in generating factual data in the medical domain, particularly medical findings related values. This evaluation also assesses whether this method could be used “on the fly” to predict detected elements not present in the final database. Since the data are numerical only, we established a prediction threshold at 20%. Predictions beyond this threshold were considered to have an accuracy score of 0, e.g., the “high” gold value for Zinc is 100 $\upmu$mol/L so value above 120 or under 80 $\upmu$mol/L are cosidered as 0. For example the high reference value for Zinc is 100 $\upmu$mol/L, so accuracy will be 1 for a prediction of 100 $\upmu$mol/L, 0.8 for a prediction of 80 or 120 $\upmu$mol/L and 0 if the prediction is above 120 or under 80 $\upmu$mol/L. Using the ChatGPT-3 language model, our method achieves a model accuracy of 78% and 80% for low and high values, respectively, with a mean based on five predictions for Electrolytes. This threshold was initially included to assess the potential of Large Language Models (LLMs) to generate the findings database. However, the final database was meticulously validated by an expert (medical doctor).

**Fig. 1:**
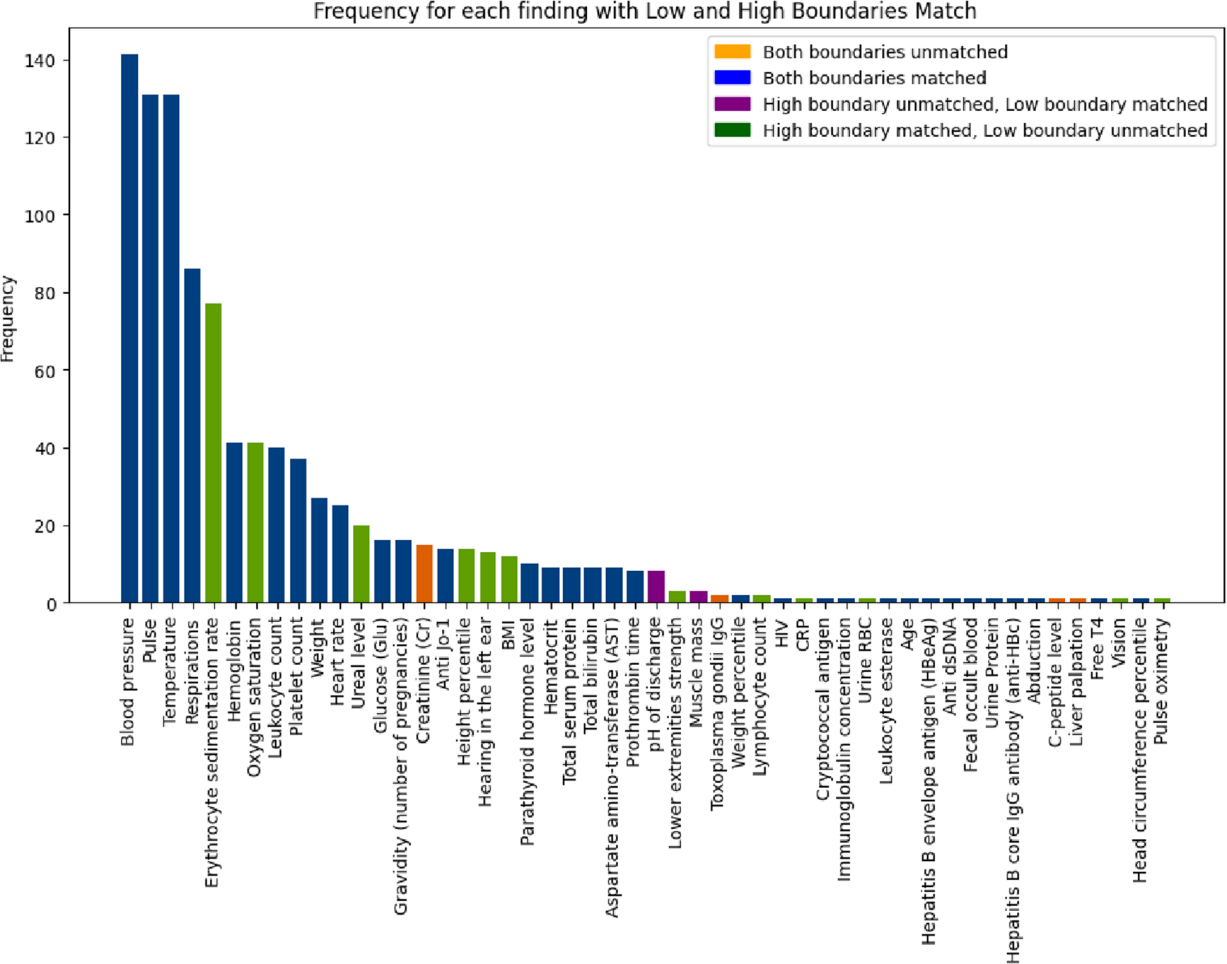
Overview of our matched findings, ordered by occurrence in our clinical cases

We experimented with both version 3.5 and 4 of the ChatGPT LLM for the semi-automatic database creation. Observing the results (Table 4), we found that both ChatGPT-3 and ChatGPT-4 showed good performance in generating boundaries values for medical findings, suggesting that these models can be reliably employed later in the proposed pipeline. It is worth noticing that the number of run predictions used to calculate the average has no impact on the result. To get some insights on the generated data, as visualized in Fig. 1, a few findings account for the majority of appearances in clinical cases.

**Table 4:** Comparison of version 3.5 and 4 of ChatGPT language models with varying runs and threshold settings, illustrating the impact on low and high accuracy metrics

LM	Run	Threshold	Low acc.	High acc.
**v-3.5**	3	20%	0.64	0.73
	3	50%	0.79	0.82
	5	20%	**0.64**	**0.79**
	5	50%	0.78	**0.84**
**v-4**	3	20%	0.63	0.72
	3	50%	0.8	0.82
	5	20%	0.63	0.73
	5	50%	**0.8**	0.81

#### Human evaluation

In order to meet the medical requirements, we employed the expertise of a clinician to evaluate a subset of our medical findings database, i.e., the 25 most frequent findings (as they appeared in our data) and 25 random findings (that were not among the 25 most frequent). The goal of this evaluation was to validate the normal range boundaries and associated medical terms for both “high” and “low” values across these 50 findings. This process yielded a total of 100 unique medical terms for validation, following the structure depicted in Table 2. This subset represents a third of our entire findings database, which has been extracted from our clinical cases.

Additionally, we provided the medical expert with a form to assess the relevance of our approach to translate medical findings into medical terms. The form contained three key questions: i) Is it medically sound and feasible to translate a finding such as “Temperature is 39^∘^C” into a medical term like “Fever,” and what are the limitations of such an approach from a medical standpoint?, ii) Are there always corresponding “high” and “low” values?, and iii) How precise should we be when defining boundary values and units? To summarize the results of the expert analysis, we concluded that i) this approach is considered to be medically accurate and relevant for the majority of the cases, except for some findings, and ii) some findings could not have both boundaries, i.e., “vision” could only be lower than normal. Finally the medical expert emphasized that iii) biological values are not strict and often associated to a shallow acceptable value. However, this is not the case in our clinical cases for student training data, in order to present clear cut cases which support the students’ training.

Following the expert’s corrections, we discovered that our database creation using the ChatGPT-4 algorithm showed a good performance, achieving an accuracy rate of 78%. An analysis of the matched findings, ordered by finding occurrence, is visualized in Fig. 1. This figure shows that errors were predominantly associated with the less represented findings, thereby highlighting a limitation of LLMs, i.e., their ineffectiveness to return knowledge from underrepresented data even in a contextual setup.

## Methods

**Fig. 2:**
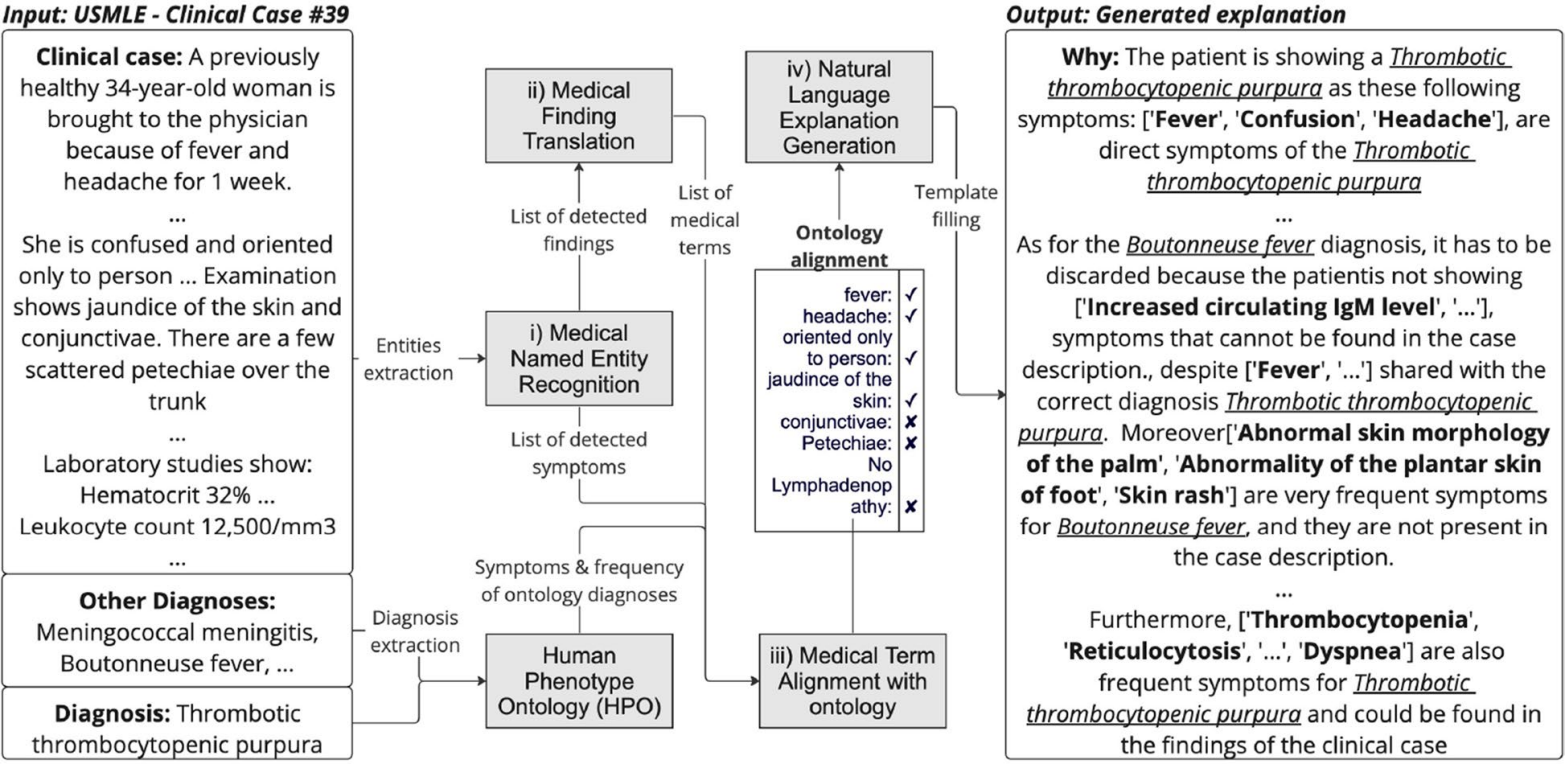
Overview of our full pipeline for symptom prediction and alignment, and NL explanation generation module. The steps are i) Medical Named Entity Recognition, ii) Medical Finding Translation, iii) Medical Term Alignment with ontology and iv) Natural Language Explanation Generation

In this paper, we aim to explain in natural language why a given diagnosis is correct or incorrect, based on a clinical case. This section presents the methods used to build our explanation pipeline^12^ based on i) Medical Named Entity Recognition, ii) Medical Finding Translation, iii) Medical Term Alignment with ontology, and iv) Natural Language Explanation Generation. The proposed architecture, called SYMEXP, is visualized in Fig. 2. This system takes a clinical case as input with the question and the correct answers to generate a natural language explanation as output. Entities are extracted from the clinical case and aligned with external knowledge to generate template-based explanations.

### Medical Named Entity Recognition: Symptoms and Findings

#### Entities detection

In order to accurately diagnose a patient’s condition, it is important to identify the symptoms that are most relevant to the possible diagnoses. This means searching through the symptoms that have been detected and reported in the clinical case, and determining which ones are most likely to be related to the patient’s condition. This can be done by considering the individual symptoms and their potential relevance to the possible diagnoses. It is also important to consider any additional information that may be available, such as the patient medical findings and other relevant factors, to be able to fully explain the diagnosis.

As introduced before, we rely on the USMLE dataset described in Resources section. In the USMLE clinical cases, layperson terms are often used, that are not detected by standard medical NER systems [58]. In order to detect also these entities, we propose a neural approach based on pre-trained Transformer Language Models, fine-tuned on manually annotated entities from our proposed MEDQA-USMLE-Symp dataset (Resources section), so as to incorporate layperson terms and findings into our training set, which are then labeled as signs or symptoms. More specifically, we cast the symptom detection problem as a sequence tagging task. Following the BIO-tagging scheme, each token is labeled as either being at the **B**eginning, **I**nside or **O**utside of a component. This translates into a sequence tagging problem with three labels, i.e., *B-Sign-or-Symptom*, *I-Sign-or-Symptom* and *Outside*.

#### Findings

**Fig. 3:**
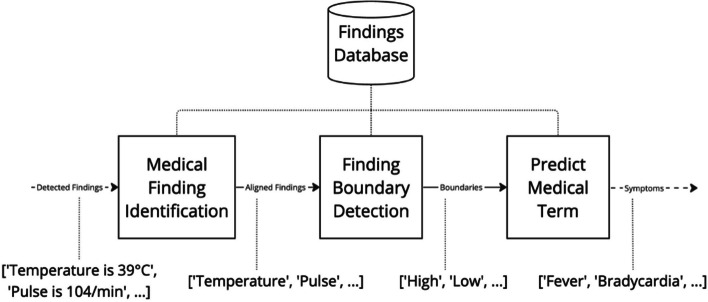
The “Findings to medical terms” module in the pipeline

While symptoms entities can be aligned with the concepts in the ontology by matching the terms identifying the symptom, findings need to be firstly pre-processed. This is why we first need to detect them via the NER, and then, to convert them, as shown in Fig. 3. Finally, both the symptoms and the medical terms (converted from detected findings) are sent to the module (iii) for alignment with the HPO ontology, as described in the next section.

Medical findings, e.g., “Platelets count is 50000 mcL”, offer important elements to the doctor to provide a diagnosis, and we aim at making reference to them in the explanations about positive and negative diagnoses. Differently from existing systems (e.g., DASH [60]), our fine-tuned NER is trained to identifying also such pieces of information. Moreover, we propose an approach to automatically convert these medical findings into medical terminology. More specifically, this module translates medical findings expressed in natural language, such as “Platelets count is 50000 mcL” into medical terms commonly found in physicians’ vocabulary or medical ontologies, for instance, “Thrombocytopenia”. To do so, we first rely on the previously created database (Resources section) and, if the term does not appear in there, we perform three key steps on the fly: (i) medical findings identification, (ii) findings boundary detection, and (iii) prediction of the associated medical term.

The first step involves accurately identifying the relevant finding within the input sentence. In our example, the finding “Platelets count” is explicitly stated, whereas in some cases, like “respirations are 22/min,” the finding may be incomplete or represented by a synonym, such as “Respiration rate” or “Breathing rate,” rather than simply “Respiration.” This step enables the alignment of the finding sentence with an entry in our database while filtering out potential errors arising from sentences that either lack findings or contain multiple findings.

The second step determines whether the detected finding value falls within normal ranges or should be classified as not applicable. If the value falls outside of the normal boundaries (from the database or predicted on the fly), we investigate whether there is an associated medical term. If the finding is not applicable, we do not proceed further.

Finally, in the third step, we predict which medical term, if any, is associated with the detected finding and boundary classification.

We proposed three methods to generate the findings knowledge by a) Input-Output (IO) Zero-Shot Prompting [84] that will serve us as a baseline, b) mimicking the doctors reasoning with a Chain of Thought (CoT) [85], and c) using Self Consistency (SC) with IO and CoT [86].

The first method, Input-Output (IO) Zero-Shot Prompting, provides a basic approach where the medical term is directly predicted from the detected finding, without any intermediate steps. This method solely relies on the capabilities of the Large Language Model (LLM) for its predictions. The second method, Chain of Thought (CoT), seeks to emulate the process of medical professionals. It divides the prediction task into two phases: firstly, determining the boundaries (both low and high) associated with the detected finding, and secondly, correlating this value range with the appropriate medical term. Lastly, the Self Consistency (SC) method enhances the decision-making process by repetitively applying the previous methods and employing a voting mechanism to select the most reliable outcome. For medical terms, this involves a count-based voting system where the most frequently occurring term is chosen. For determining value boundaries, we experimented with two approaches, i.e., an average on all predictions, and a count-based voting system.

The converted findings are then injected together with the detected symptoms into the medical term alignment algorithm. This will ensure the inclusion of findings interpretation within the generated explanations in the last step of the pipeline (Fig. 2).

### Medical term alignment

The medical term alignment module (Fig. 2) associates, whenever possible, the pertinent symptoms or translated findings mentioned in the clinical case description with a term of a diagnosis found in the HPO knowledge base. The proposed framework consists of two different steps, where: *(a)* we retrieve from HPO the required diagnosis information (i.e., the symptoms and how common they are), then the terms in the case are detected and extracted using the modules introduced in the previous section; *(b)* the relevancy of each symptom is assessed by matching the detected medical term with the ones retrieved from HPO, e.g., “Platelets count is 50000 mcL” to HP:0001873^13^ (Thrombocytopenia). The matched terms are then used to generate natural language argument-based explanations for correct and incorrect diagnoses.

Regarding the matching module, we experimented with two different methods to align our detected entities with terms in HPO by *(i)* directly comparing the computed embeddings of the detected entities with the embeddings of the terms in HPO, and *(ii)* by taking into account the context in which the entities are detected and applying the same context to every term in HPO. The reasoning behind the latter is that the corresponding entities in HPO should not change the semantics of the sentence with respect to the other symptoms. To align our detected symptoms and converted findings with the equivalent HPO terms, we calculate the cosine distance of each embedding of the HPO terms with respect to the embedding of the detected symptom.

It is worth noticing that, for task *(ii)*, it is necessary to calculate the context embeddings “on the fly” because each context is unique and depends on the clinical case in which it has been detected. However, to avoid recomputing all HPO term embeddings on the fly for each new context (since the ontology contains 10,319 unique terms), we propose to generate all the HPO terms embedding at once and store them. Therefore, this module takes as input the symptoms and translated findings detected by the previous module and looks for the context^14^ of these symptoms in the clinical case.

The context *C* is embedded using sentence embedding methods and saved separately from the symptoms *S*, and the two embeddings are merged together ($C + S$) to form the reference *R*. This same context embedding *C* is added in the same way to each HPO term embedding $T_1, T_2, \dots , T_i$ to form the candidates $C_1, C_2, \dots , C_i$. We compute and retrieve the five best cosine distances between *C* and *R* to address a fair comparison with the other systems.

### Explanation generation

We propose a template-based explanation generation module based solely on the symptoms and findings that are relevant to explain the diagnosis. To do this we propose several templates that tackle different kinds of explanations, going from explaining why a patient was given a certain diagnosis, to explaining why the alternatives cannot be considered as viable options. We support our explanations with statistical information obtained from HPO such as the frequency of each symptom incidence, and we propose to look for possible symptoms that were not detected by the system but are frequent for a certain disease. These explanations are made from the aligned detected terms in the ontology, keeping the references with original words in the clinical cases (i.e., laypersons symptoms and patient tests or analysis). The detailed templates and examples are described in Generating Natural Language Explanations section.

## Experiments

In this section, we report on the experimental setting, the obtained results and the error analysis for the named entities detection, the finding translation and the symptom alignment methods. It is worth noticing that the presented model can be applied also to different kinds of clinical cases, ensuring the generalisability of the proposed approach.

### Experimental setting

#### Medical entity detection

For the entity detection task, we experiment with different transformer-based Language Models such as BERT [87], SciBERT [53], BioBERT [54], PubMedBERT [55] and UmlsBERT [57] initialized with their respective pre-trained weights. All the models we employ are specialized in the biomedical domain, with the exception of BERT which will serve us as a baseline. To fine-tune the LMs, we use the PyTorch implementation of Huggingface [88] (v4.18). For BERT, we use the uncased base model with 12 transformer blocks, a hidden size of 768, 12 attention heads, and a learning rate of 2.5e-5 with Adam optimizer for 3 epochs. The same configuration was used to fine-tune SciBERT BioBERT, PubMedBERT and UmlsBERT. For SciBERT, we use both the cased and uncased versions, and for BioBERT we use version 1.2. Batch size was 8 with a maximum sequence length of 128 subword tokens per input example. Both the dataset and the guidelines used to train our NER model are available in this project repository https://github.com/Wimmics/MEDQA-USMLE-Symp.

#### Finding converter

In our experiments, we adopted the latest large generative language model from OpenAI, ChatGPT gpt-3.5-turbo-0301 and gpt-4 [82, 83]. We employed the snapshot of gpt-3.5-turbo from March 1st, 2023. This model was used for both joint and combined baselines, employing classic handcrafted prompts available in Appendix 9.

For the CoT steps that mimics doctors’ reasoning, we used the FuzzyWuzzy^15^ Python package version 0.18.0 for the task of medical finding identification. This package leverages the Levenshtein Distance to calculate the differences between sequences in a user-friendly package. Concerning the finding values detection using string matching, we employed a Python regular expression with the regex package version 2022.10.31 : 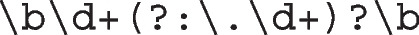


We experiment as an alternative a NER approach, using med7 [89] with the “en_core_med7_lg” model, trained on MIMIC-III free-text electronic health records, and Spacy version 3.5.2. All experiments were conducted using Python 3.10.11 directly in a Google Co-Laboratory Pro notebook. The medical finding database, validated by medical expert is available in our project repository^16^.

#### Ontology alignment

Regarding the matching module, we experimented with two different methods to align the detected entities with the terms in HPO by *(i)* directly comparing the computed embeddings of the detected entities with the embeddings of the terms in HPO, and *(ii)* by taking into account the context in which the entities are detected and applying the same context to every term in HPO. In the experimental setting of both tasks (i) and *(ii)*, we use the static pre-trained embeddings GloVe 6B as well as BERT, SciBERT, BioBERT and UmlsBERT with the same configurations as in the medical NER task. Each embedding is calculated with Sentence Transformer Document Embeddings using the flair framework [90], using the same Python environment as the previous modules.

We defined a test set of 23 cases from the MEDQA-USMLE-Symp dataset (Resources section) where *(i)* we retrieved from HPO the symptoms related to the diagnoses for each case, and *(ii)* we manually aligned the annotated symptoms in the case to the concepts from HPO. This resulted in 162 symptoms aligned to a specific term in HPO that serve us as a testing set for our matching module.

As mentioned in Background section, the system proposed by [60] offers a similar approach to translating layperson terms to medical terms in HPO. However, their work does not take into account the context in which a symptom is mentioned in the text. To compare with this approach and due to the unavailability of their model, we rely on their online demo, which outputs only the top 5 ranking of the HPO terms that are closest to the input symptom. To perform a comparison with our pipeline, we first compute the accuracy of the aligned symptoms using our symptom alignment module and then replaced it with Manzini et al. [60] proposed system (DASH). Results are shown in Table 9.

Since a symptom can be composed of several words (e.g., “shortness of breath”), we split the symptom into words that we encode by either using each word as an input on Glove [91], or extracting directly from the contextualized models the representation of the symptom by summarizing the hidden states of the last four layers in the model. We then sum the vectors of each word to get an n-gram representation of the symptom. We also explore sentence embeddings, by making use of Sentence-BERT [92], a new model that derives semantically meaningful sentence embeddings (i.e., semantically similar sentences are close in vector space) that can be compared using cosine similarity. Sentence-BERT can be used with different pre-trained models, in this work we focus on the models BERT [87], SciBERT [53], UMLSBERT [57] and S-PubMedBert by [93]. The first represents a competitive baseline in our experiments since it is the SOTA model for comparing sentences cross-domain, while the three latter models are pre-trained on scientific or medical data or both.

To tackle both tasks, we make use of our annotated dataset (Resources section). The annotations are converted into two datasets, one for each part of the pipeline. The first dataset is used for the symptom detection task, and it is in the CoNLL format for token-wise labels. The second dataset, for the symptom alignment task, is converted into a csv format, where each symptom in the clinical case description and available related knowledge (i.e., the list of symptoms and their frequencies for each possible diagnosis associated with the case) extracted from HPO are paired. Finally, we rely on the HPO ontology, utilizing the requests package version 2.27.1 and the public HPO endpoint https://hpo.jax.org/api/hpo/search.

### Results

In the following section, we report on the results obtained for our pipeline presented in Fig. 2, as well as the error analysis.

#### Medical NER

As introduced before, the first task addressed in our pipeline is to detect the medical named entities. The results for the symptom detection task are shown in Table 5 in macro multi-class precision, recall, and F1 score. We can observe that all models perform similarly, with the best results from the specialized SciBERT [53] model. The biggest difference in performance is given by comparing SciBERT uncased with PubMedBERT, with the SciBERT model performing better. Interestingly, BERT performs closely to the specialized models, and, in some cases, it outperforms them. This may be due to the fact that the clinical cases from our dataset are written for medical exams at the med school. They contain some technical specialized words, but overall the symptoms are described in layperson terms. It is also worth noticing that the majority of our labels do not pertain to medical terminology (e.g. Age and Population Group, Location and Temporal Concept). Sign or Symptom and Finding are the only labels that require specialized vocabulary.

**Table 5:** Results for entity recognition in macro multi-class precision, recall, and F1-score

Model	P	R	F1
BERT	0.85	0.84	0.84
BioBERT v1.2	0.84	0.85	0.84
UmlsBERT	0.85	0.85	0.85
PubMedBERTbase	0.83	0.84	0.83
SciBERT cased	0.85	0.85	0.85
SciBERT uncased	**0.85**	**0.86**	**0.86**

Overall, SciBERT uncased is the best-performing model (in bold) with a macro F1-score of 0.86, outperforming the other approaches for each of the categories. In Table 6 and Table 7 we report on the performances for each entity with the best-performing models SciBERT and BERT. The *Sign or Symptom* detection task obtains a 0.82 F1 score. In the work of [94], the authors also detect symptoms obtaining an F1 score of 0.61. However, these results can not be directly compared since the datasets on which both models were fine-tuned are different: we train on clinical cases, while they use dialogues between doctors and patients. Moreover, given that the dataset they use is not released, we can not evaluate our approach on their data to compare the results.

**Table 6:** Results for entity recognition using our best performing model (SciBERT uncased) in P, R, and F1-score

Entity	P	R	F1
Other	0.93	0.91	0.92
Age Group	1.00	0.97	0.98
Finding	0.85	0.88	0.86
Location	0.74	0.80	0.77
No Symptom Occurrence	0.79	0.72	0.75
Population Group	0.88	0.95	0.91
Sign or Symptom	0.83	0.82	0.82
Temporal Concept	0.78	0.87	0.82
Weighted avg	0.89	0.89	0.89
Macro avg	0.85	0.86	0.86

**Table 7:** Results for entity recognition using BERT uncased in P, R, and F1-score

Entity	P	R	F1
Other	0.92	0.91	0.92
Age Group	1.00	0.97	0.98
Finding	0.87	0.87	0.87
Location	0.74	0.80	0.77
No Symptom Occurrence	0.79	0.72	0.75
Population Group	0.88	0.95	0.91
Sign or Symptom	0.83	0.82	0.82
Temporal Concept	0.78	0.87	0.82
Weighted avg	0.89	0.89	0.89
Macro avg	0.85	0.86	0.86

Raza et al. [58] proposed a transformer-based NER system employing DistillBERT [59] that is able to recognize a wide range of clinical entity types, encompassing medical risk factors, vital signs, drugs, and biological functions. Their approach, which primarily relies on the Case Report dataset MACROBAT [95], focuses on doctors vocabulary. To make a fair comparison, we evaluated the output of their model, BioEN, at a token level using our own test set, specifically comparing the accuracy of the sign_or_symptoms labels. The results highlight a significant gap between the two approaches in terms of performance: out of 285 gold tokens, BioEN detected only 79, whereas our model identified 260. This disparity is primarily due to our specific focus on the detection of data encoded in layperson vocabulary.

#### Finding converter

Here we describe the results of the prediction task of the medical terms associated to the detected findings (Resources section). The efficacy of our medical finding to medical term on-the-fly conversion module is detailed in Table 8. Table 8 presents the accuracy of our Finding Converter module in identifying boundaries, both in terms of values and associated terms. The mentioned 78% accuracy refers to the accuracy achieved in determining values, considering the previously discussed 20% threshold. The accuracy figures are computed based on the final version of the generated database, which achieved an accuracy of 78% for “low” boundaries (88%) and “high” boundaries (68%), after the doctor validation. The proficiency of the model in predicting “low” boundaries could be attributed to their higher frequency and often singular appearance as the defining boundary for a medical finding. For instance, the “vision” finding exemplifies this trend, as it only has a “low” boundary, represented by “blindness”, with no corresponding “high” boundary. The context added by the intermediate steps seems to fine-tune the language model’s knowledge and aids in generating more suitable responses. The Self-Consistency method does not improve the results.

**Table 8:** Results for on-the-fly findings to medical terms prediction using the generative LLM ChatGPT

Prompting Method	Accuracy
IO ChatGPT 4	0.64
IO ChatGPT 3	0.52
CoT ChatGPT 4	**0.66**
CoT ChatGPT 3	0.52
SC IO ChatGPT 4	0.64
SC IO ChatGPT 3	0.54
SC CoT ChatGPT 4	**0.66**
SC CoT ChatGPT 3	0.54

The results of the symptom alignment module, that aim to associate the detected entities in the clinical case with the HPO ontology, are summarised in Table 9. As baseline models, we propose to use the same methods but without the context of the symptoms, similarly to *DASH* [60]. In Table 9, we show only the best-performing baseline *PubMedBERT no context* obtaining similar results to *DASH* (0.41 and 0.37, respectively). Adding contextual representation to the embeddings results in a significant improvement (up to 0.70 in accuracy) supporting the hypothesis that context plays an important role when translating layperson terms to formal medical terms.

**Table 9:** Results for DASH and our symptom alignment method using different embeddings with and without context (accuracy score)

Model	Accuracy
DASH	0.37
BERT + no context	0.39
SciBERT + no context	0.39
UMLSBERT + no context	0.44
S-PubMedBERT no context	0.53
BERT + context	0.53
SciBERT + context	0.57
UMLSBERT + context	0.59
S-PubMedBERT + context	**0.70**

### Error Analysis

The main limitation of adopting HPO as medical knowledge base concerns the number of symptoms associated with each diagnosis. For some diagnoses, we have multiple symptoms, while for others we can have only one or none. We notice that in those cases where the diagnosis is a mental disease, the model tends to make more mistakes. Inspecting HPO for this kind of diagnoses, we find that either the diagnosis does not appear in the HPO ontology or the symptoms tend to be more general, including a lot of common symptoms like *changes in appetite* or *low energy*, that alone may not be relevant but all together indicate a precise diagnosis. Moreover, some relevant symptoms may not be described explicitly but encoded in the clinical cases as *Findings*. These findings, even translated into a medical term, do not appear in the symptoms list extracted from HPO since this ontology focuses on pathological terms. The finding “Gravidity” (i.e., number of pregnancies) exemplifies this insight because being “Multiparous” is not pathological but is the medical term associated to the “high” boundary of the finding. Therefore it would be useful for the explanation but it does not match in our system because of the HPO limitations. Moreover, a diagnostic can be supported by a less specific interpretation of a finding, e.g., the thrombotic thrombocytopenic purpura can be explained by a patient arrhythmia defined as “A irregular heartbeat / A problem with the rate or rhythm of your heartbeat” but our system will detect either a Bradypnea or a Tachypnea that are both kinds of Arrhythmia. A possible extension of this work consists in a deeper investigation of the ontologies to find a way to align with different granularity the detected finding.

Given that we rely on HPO only, some diseases or diagnoses are not present in the knowledge base, preventing us to generate the associated explanations. Combining HPO with more specialized medical knowledge bases is a future direction for this work, both to complete the information we have, and also to integrate new diagnoses.

## Generating Natural Language Explanations

In the previous section, we described the first steps of our pipeline for automatically identifying the relevant symptoms and findings which occur in the clinical case description and then matching them with the terms associated with the diseases in the medical knowledge base HPO. We move now to the last step of the pipeline, i.e., the generation of natural language explanatory arguments, according to the identified relevant symptoms and findings for the correct and incorrect diagnoses. We denote a lot of different methods to tackle the argumentative explanation generation, mostly approached by generative neural architecture like Recurrent Neural Network, GPT or T5 [65, 67, 69, 96, 97]. Given the specificity of the clinical data we are dealing with, and the limitations of such generative approaches (hallucinations, bias) [98], we decided to address this task by generating explanations through the definition of explanatory patterns [71–73]. We have therefore defined different patterns which take into account the different requirements of our use case scenario, where we aim at *(i)* explaining the correct answer by the detected symptoms/findings and their frequency, *(ii)* explaining why the incorrect options cannot hold, and *(iii)* highlighting the relevant symptoms not explicitly mentioned in the clinical case. Let us consider the following clinical case, where in bold we highlight the **symptoms**, in italic the *findings* and we underline the relevant symptoms and findings supporting the correct answer.

### Clinical case

A previously healthy 34-year-old woman is brought to the physician because of **fever** and **headache** for 1 week. She has not been exposed to any disease. She takes no medications. Her *temperature is 39.3°C (102.8°F)*, *pulse is 104/min*, *respirations are 24/min*, and *blood pressure is 135/88 mm Hg*. She is **confused** and **oriented only to person**. Examination shows **jaundice of the skin** and **conjunctivae**. There are a few scattered **petechiae** over the trunk and back. There is **no lymphadenopathy**. Physical and neurologic examinations show **no other abnormalities**. *Test of the stool for occult blood is positive*. Laboratory studies show: *Hematocrit 32% with fragmented and nucleated erythrocytes Leukocyte count 12,500/mm3* *Platelet count 20,000/mm3* *Prothrombin time 10 sec* *Partial thromboplastin time 30 sec* *Fibrin split products negative* *Serum Urea nitrogen 35 mg/dL* *Creatinine 3.0 mg/dL* *Bilirubin Total 3.0 mg/dL* *Direct 0.5 mg/dL* *Lactate dehydrogenase 1000 U/L* *Blood and urine cultures are negative*. A CT scan of the head shows **no abnormalities**. Which of the following is the most likely diagnosis?

This example is extracted from the MEDQA-USMLE-Symp dataset and the (already known) correct diagnosis is Thrombotic thrombocytopenic purpura, whilst the other (incorrect) options are Disseminated intravascular coagulation, Immune thrombocytopenic purpura, Meningococcal meningitis, Sarcoidosis and Systemic lupus erythematosus.

### Why Pattern

We focus here on the correct diagnosis explanation pattern, which allows explaining why this is the correct diagnosis. We define the following template to generate our natural language explanations:

#### Definition 1

(Why for correct diagnosis) The patient is showing a [CORRECT DIAGNOSIS] as these following symptoms [**PERFECT MATCHED SYMPTOMS**, **MATCHED SYMPTOMS**, **MATCHED FINDINGS**] are direct symptoms of [CORRECT DIAGNOSIS].

Moreover, [**OBLIGATORY SYMPTOMS**] are obligatory symptoms (always present, i.e., in 100% of the cases) and [**VERY FREQUENT SYMPTOMS**] are very frequent symptoms (holding on 80% to 99% of the cases) for [CORRECT DIAGNOSIS] and are present in the case description.^17^

In Template 1, the [CORRECT DIAGNOSIS] represents the correct answer to the question “Which of the following is the most likely diagnosis?” and therefore the correct diagnosis of the described disease. The [**SYMPTOMS**] / [**FINDINGS**] in bold represent the medical terms automatically detected through the first module of our pipeline, and they are also underlined when they are considered as relevant by our matching module, i.e., they are listed among the symptoms for the disease in the HPO knowledge base. Both [**PERFECT MATCHED SYMPTOMS**] and [**MATCHED SYMPTOMS**] in Template 1 are considered relevant but they differ in the confidence level the system assigns to the matched symptoms. This allows us to integrate a notion of granularity in our explanations and to rely on the symptoms or raw findings detected in the clinical case that strongly match with a symptom in HPO. If the system does not detect any relevant symptom, no explanation is generated for the correct answer. Furthermore, we employ the information about the symptom frequencies (retrieved through HPO) in the [**OBLIGATORY SYMPTOMS**] and [**VERY FREQUENT SYMPTOMS**] to generate stronger evidence to support our natural language argumentative explanations. Sometimes the frequencies are not available in the HPO, in which case we do not display them in our final explanation.

We present now some examples of explanatory arguments automatically generated by our system.

#### Example 4

The patient is showing a [Thrombotic thrombocytopenic purpura] as these following symptoms [**Headache**, **Fever**, **Confusion (Oriented to persons)**, **Thrombocytopenia (Platelet count 20,000/mm3)**, **Reticulocytosis (Jaundice of the** **skin)** and **Decreased serum creatinine (Creatinine 3.0 mg/dL)**] are direct symptoms of [Thrombotic thrombocytopenic purpura].

Moreover [**Reticulocytosis (Jaundice of the skin)** and **Thrombocytopenia (Platelet count 20,000/mm3)**] are very frequent symptoms (holding on 80% to 99% of the cases) for [Thrombotic thrombocytopenic purpura] and are present in the case description.

When filling the [**SYMPTOMS** and **FINDINGS**] span in Template 1, we inject only the terms matched in the HPO for the [**PERFECT MATCHED SYMPTOMS**], and we combine the HPO with the detected symptoms and findings in the case description for the [**MATCHED SYMPTOMS** and **MATCHED FINDINGS**] in this form: [**matched term in HPO** (***detected term in the clinical case***)] (e.g., in Example 4: **Confusion (Oriented to persons)** and **Thrombocytopenia (Platelet count 20,000/mm3)**)

### Why not Template

Explaining why a diagnosis is the correct one is important, but it is also necessary to be able to say why the other options are not correct as possible diagnoses for the clinical case under investigation [99]. We, therefore, propose to provide explanations based on the relevant symptoms for the incorrect options by contrasting them with the clinical case at hand.

#### Definition 2

(Why not for incorrect diagnosis) Concerning the [INCORRECT DIAGNOSIS] diagnosis, it has to be discarded because the patient in the case description is not showing [**INCORRECT DIAGNOSIS SYMPTOMS / FINDINGS FROM HPO (MINUS DETECTED SYMPTOMS IN CASE**)] symptoms.

Despite [**SHARED CORRECT SYMPTOMS / FINDINGS**] symptoms shared with the [CORRECT DIAGNOSIS] correct diagnosis, the [INCORRECT DIAGNOSIS] diagnosis is based on [**INCORRECT DIAGNOSIS SYMPTOMS**].

Moreover, [**OBLIGATORY SYMPTOMS**] are obligatory symptoms (always present, i.e., in 100% of the cases) and [**VERY FREQUENT SYMPTOMS**] are very frequent symptoms (holding on 80% to 99% of the cases) for [INCORRECT DIAGNOSIS], and they are not present in the case description.

Template 2 can be applied to each incorrect possible answer of the case, individually. The incorrect answer corresponds to the [INCORRECT DIAGNOSIS] and [**INCORRECT DIAGNOSIS SYMPTOMS / FINDINGS**] are all relevant terms associated with this disease in the HPO knowledge base, without the terms in common with the correct answer. Again, in the template, we use the frequencies provided by HPO to provide further evidence to make our explanatory arguments more effective. The template includes therefore with [**OBLIGATORY SYMPTOMS**] and [VERY FREQUENT SYMPTOMS] the mandatory and very frequent symptoms of the incorrect diagnosis, which are missing in the clinical case description. The following explanations are automatically generated for (one of) the incorrect diagnoses of the clinical case we introduced at the beginning of this section.

#### Example 5

Concerning the [Meningococcal meningitis] diagnostic, it has to be discarded because the patient in the case description is not showing [**Stiff neck, Nuchal rigidity or CSF pleocytosis, Increased CSF protein, Hypoglycorrhachia**] symptoms.

Despite [**Petechiae, Fever, Headache**] symptoms shared with the [Thrombotic thrombocytopenic purpura] correct diagnosis, the [Meningococcal meningitis] diagnosis is based on [**Stiff neck, Nuchal rigidity or CSF pleocytosis, Increased CSF protein and Hypoglycorrhachia**].

Moreover, [**Stiff neck, Nuchal rigidity, CSF pleocytosis, Increased CSF protein or Hypoglycorrhachia**] are very frequent symptoms (holding on 80% to 99% of the cases) for [Meningococcal meningitis] and are not present in the case description.

Example 5 shows the natural language explanation of why the possible answer [Meningococcal meningitis] is not the correct diagnosis given the symptoms discussed in the clinical case description. In case the disease is not found in HPO, we do not generate the associated explanation.

### Additional Explanatory Arguments

In order to enrich our explanations with additional explanatory arguments to improve critical thinking in the medical residents, we also generate another template. Indeed, in some clinical cases, it is possible that the detected terms are not sufficient to explain the diagnosis or sometimes the informations are missed by the proposed system.

In some situations, our system is not able to abstract some findings that are important for the diagnosis as for the Thrombotic thrombocytopenic purpura, a Very frequent symptom is Arrhythmia, defined as “Any cardiac rhythm other than the normal sinus rhythm”. Our system will detect a Tachycardia that, by definition is a kind of Arrhythmia. Template 3 aims at drawing the medical residents’ attention to (statistically) important symptoms that are missing or not explicitly mentioned in the clinical case description:

#### Definition 3

Furthermore, [**CORRECT DIAGNOSIS VERY FREQUENT TERMS (MINUS MATCHED TERMS)**] are also frequent symptoms for [CORRECT DIAGNOSIS] and could be found in the findings of the clinical case.

Example 6 is generated by our system and brings attention to Arrhythmia. This additional explanatory argument complements the explanation we generate for the correct and incorrect diagnoses in the case presented at the beginning of this section.

#### Example 6

Furthermore, [**Arrhythmia, Generalized muscle weakness**, and **Microangiopathic hemolytic anemia**] are also frequent symptoms for [Thrombotic thrombocytopenic purpura] and could be found in the findings of the clinical case.

## Discussion and concluding remarks

The pipeline presented in this paper aims to generate template-based natural language explanations to argument from a symptomatic point of view why a diagnosis is correct and why the remaining ones are incorrect. More precisely, based on two novel annotated linguistic resources, our pipeline *(i)* automatically identifies in a clinical case description the relevant symptoms and matches them to the HPO medical knowledge base terms to associate symptoms to the correct and incorrect diagnoses proposed as potential answers to the test, *(ii)* automatically identifies in the case description the main findings and associate them to medical terms and biological boundaries in order to assess their role with respect to the correct and incorrect diagnoses, and *(iii)* automatically generates pattern-based natural language explanatory arguments highlighting *why* a certain answer is the correct diagnoses and *why* the others are not. Extensive experiments on a dataset of 314 clinical cases in English on various diseases show good results (0.86 on symptom detection and, 0.56 and 0.70 on relevant symptom matching for Top 1 and Top 5 matches respectively, and 0.78 on findings), outperforming competitive baselines and SOTA approaches. Given the sensibility of the medical domain and the fact that this system is intended as an example of AI in education and training, our explanations have a didactic goal which is exemplified through the enrichment of the data available in the clinical case description with further verified information from the knowledge base. In our work we have decided to adopt a method based on templates to generate explanations in order to avoid any hallucination problems associated with LLMs. Although this approach has its own limitations, such as being design-dependent, it provides a robust and verified strategy which is more suitable to the medical domain.

Several future work lines arise from this work. First, we plan to address a user evaluation with med residents. Even though clinical doctors have been involved in the definition of the annotation guidelines, a user evaluation with med residents is required to get their feedback on our explanatory arguments. Second, we plan to focus on user-specific explanations, adapting the language and level of detail depending on whether the user is a doctor, a resident, or a potential generic user. Another relevant future research line consists in the adoption of further medical ontologies to reduce the HPO limitations and enhance the performances. It would also be interesting to explore the relationships between ontology entities to improve the detection of symptoms, and therefore the generated explanations. For instance, one of the frequent errors in our experiments concerns cardiac arrhythmia, often a symptom linked to a diagnosis in ontologies but difficult to detect because it is often expressed “too” precisely via the symptoms “tachycardia” or “bradycardia”, which are both forms of cardiac arrhythmia (low or high rhythm). Moreover, given the difference on our performances to predict low and high boundaries (88% and 68%, respectively), it may be interesting to address further experiments, comparing our proposed automated methods and re-submit our results to undergo another expert evaluation. Finally, we plan to make these explanations interactive to address a rule-based dialogue with the student in order to focus on precise aspects of the clinical case and go into more precise or generic explanations if required by the student.

## Data Availability

All parameters to allow the reproducibility of the experiments are available in section 5. The dataset containing the NER training data and the database of medical findings are available on the following repo: https://github.com/Wimmics/MEDQA-USMLE-Symp.

## Findings converter experiments prompts

This appendice shows the prompts used in our findings converter experiments. The label *[FINDING]* is replaced on the fly by the current finding name.

## Abbreviations

NL: Natural language
UMLS: Unified medical language system
XAI: Explanatory artificial intelligence
LLM: Large language model
SOTA: State of the art
NER: Named entity recognition
NEs: Named entities
NLP: Natural language processing
AI: Artificial intelligence
HPO: The human phenotype ontology
KB: Knowledge base
PoS: Part of speech
BioEN: Bio-epidemiology-NER
EMR: Electronic medical records
EHR: Electronic health records
USMLE: United state medical board examination
MCMLE: Mainland China medical board examination
TWMLE: Taiwan medical board examination
CUI: Concept unique identifiers
IAA: Inter annotator agreement
ICD: International classification of diseases
IE: Information extraction
NLI: Natural language inference
RQE: Recognizing question entailment
QA: Question answering
WHO: World health organization
Glu: Glucose
IO: Input output
CoT: Chain of thoughts
SC: Self consistency
BIO: Beginning inside outside

## Footnotes

1 https://www.snomed.org/value-of-snomedct.

2 https://github.com/Wimmics/MEDQA-USMLE-Symp

3 https://github.com/Wimmics/MEDQA-USMLE-Symp

4 https://ir.cs.georgetown.edu/resources/quick-umls.html

5 https://github.com/Wimmics/MEDQA-USMLE-Symp

6 https://github.com/Wimmics/MEDQA-USMLE-Symp

7 https://hpo.jax.org/app/browse/term/HP:0040279

8 https://www.orpha.net/consor/cgi-bin/index.php?lng=FR

9 https://www.omim.org/

10 Medscape: https://emedicine.medscape.com/article/2172316-overview

11 https://emedicine.medscape.com/article/2172316-overview

12 In this paper, we do not report any experiment carried out on live vertebrate (or higher invertebrates), humans or human samples. As we rely on standard benchmarks in the field of AI in medicine, it is not possible to identify patient/participant information as the clinical cases are not real cases but they are explicitly conceived for training medical residents. Our research does not concern either human transplantation research, nor it reports results of a clinical trial.

13 https://hpo.jax.org/app/browse/term/HP:0001873

14 The context consists of the sentence(s) containing the symptom and the entire clinical case.

15 https://pypi.org/project/fuzzywuzzy/

16 https://github.com/Wimmics/MEDQA-USMLE-Symp

17 Sources from HPO: https://hpo.jax.org/app/browse/term/HP:0040279
